# Influence of Different Implantoplasty Designs on the Fatigue Resistance of Dental Implants: A Systematic Review

**DOI:** 10.3390/jcm14176103

**Published:** 2025-08-29

**Authors:** Manuel León Velastegui, Rubén Agustín-Panadero, Aitana Rico-Coderch, José Amengual-Lorenzo, Carlos Labaig-Rueda, María Fernanda Solá-Ruiz

**Affiliations:** 1 School of Dentistry, Faculty of Health Sciences, Universidad Nacional de Chimborazo, Riobamba 06103, Ecuador; maleon@unach.edu.ec; 2 Department of Stomatology, Faculty of Medicine and Dentistry, University of Valencia, 46010 Valencia, Spain; ruben.agustin@un.es (R.A.-P.); jose.amengual@uv.es (J.A.-L.); carlos.labaig@uv.es (C.L.-R.); m.fernanda.sola@uv.es (M.F.S.-R.)

**Keywords:** implantoplasty, fracture resistance, dental implant

## Abstract

**Objectives**: To analyze the impact of implantoplasty on the mechanical resistance of dental implants, considering different implantoplasty designs and implant types. **Methods**: A systematic review was conducted in accordance with PRISMA guidelines. A search was performed in four databases: PubMed, Scopus, Web of Science, and Embase, along with a manual search for additional relevant studies. In vitro studies assessing the mechanical resistance of dental implants subjected to implantoplasty were included. A total of 136 studies were identified; after duplicate removal using Rayyan, and screening by title and abstract, 17 studies were ultimately selected after full-text assessment. **Results**: In vitro studies on external hexagon implants showed that fracture resistance in control groups ranged from 773.1 N to 1660 N for implants with a 4 mm diameter, and from 478.1 N to 1650 N after implantoplasty. For 3.5 mm diameter implants, values ranged from 548.8 N to 1276.1 N in control groups, and from 465.9 N to 1211.7 N after implantoplasty. In internal hexagon connections, fracture resistance after implantoplasty ranged between 321.7 N and 739 N. Conical connections exhibited a broader range of resistance values after implantoplasty, from 315.9 N to 2395.3 N. **Conclusions**: Implantoplasty reduces the mechanical strength of dental implants. Increased implantoplasty length correlates with decreased resistance, particularly affecting narrow implants. The prosthetic connection most affected by this procedure is the external hexagon, followed by the internal hexagon, with the conical connection being the most resistant.

## 1. Introduction

Dental implants have become a reliable and predictable solution for the rehabilitation of edentulous patients or those with partial tooth loss. However, biological complications such as peri-implantitis, a progressive and irreversible condition [1,2], pose a significant challenge to the long-term success of implant treatments. Peri-implantitis is characterized by inflammation, bleeding, and suppuration of peri-implant tissues, along with progressive bone loss around the implant [2,3,4], which can compromise its stability and functionality.

Implantoplasty, an adjunctive surgical therapy, is recommended for supracrestal bone defects, horizontal bone loss with exposed threads in non-aesthetic areas, depending on patient needs and satisfaction [5]. This procedure has been proposed as an effective procedure to reduce bacterial load on the implant surface and improve peri-implant tissue integration. It involves mechanical removal of the implant threads and rough surface [6], thereby smoothing the surface and reducing bacterial adhesion [7]. Various techniques and instruments can be used, which favor reduced bacterial colonization, promote fibroblast growth, and enhance healing [8,9,10,11,12]. Nevertheless, concerns remain regarding the effect of implantoplasty on implant structural integrity and the surrounding tissues.

Implantoplasty thins the implant walls and adversely affects their mechanical resistance, which depends on several factors, including implant diameter, platform design, and exposure to functional loads [13]. The removal of material during implantoplasty may compromise the structural integrity of the implant, reduce its load-bearing capacity, and increase the risk of fractures and long-term mechanical failure [14]. Additionally, it may cause overheating [10,15] and lead to harmful titanium particle deposition in surrounding tissues [15], which is further exacerbated by implant corrosion [16].

The objective of this study is to evaluate the effect of implantoplasty on the mechanical integrity of dental implants. Although several systematic reviews have addressed this topic, none have comprehensively assessed all the parameters considered in the present work. Accordingly, this review focuses on the available literature that includes experimental analyses of fracture and fatigue resistance before and after implantoplasty. This systematic review aims to provide a more thorough and integrated perspective, with the goal of determining whether implantoplasty has a significant impact on the structural and biomechanical integrity of dental implants.

## 2. Materials and Methods

### 2.1. Protocol and Registration

A systematic literature review was conducted in accordance with the PRISMA (Preferred Reporting Items for Systematic Reviews and Meta-Analyses) 2020 checklist [17]. The review protocol was registered in the Open Science Framework under the registration: https://doi.org/10.17605/OSF.IO/TU98A, accesed on 28 May 2025.

### 2.2. Research Question

The research question was formulated using the PICO framework (Patient, Intervention, Comparison, Outcome):

What is the fracture resistance of dental implants with peri-implantitis subjected to implantoplasty?

- Population: Dental implants with peri-implantitis
- Intervention: Implantoplasty
- Comparison: Untreated dental implant
- Outcome: Fracture resistance

### 2.3. Outcome Measures

The outcome measures were the fracture resistance and cyclic fatigue resistance of dental implants subjected to implantoplasty, a mechanical process of removing the threads and surface roughness to prevent bacterial plaque accumulation. The measurements were performed using a universal testing machine, according to ISO 14801:2007 and 2017 standards [18].

### 2.4. Eligibility Criteria

- Inclusion criteria: In vitro studies involving dental implants subjected to implantoplasty, and studies performing fatigue tests without limitation of diameter and length of dental implants.
- Exclusion criteria: In vivo studies, Randomized clinical trials, in silico studies, orthodontic implants, case reports.

### 2.5. Information Sources and Search Strategy

The databases searched up to March 2025 included PubMed, Embase, Scopus, and Web of Science. The search strategy used Boolean operators (AND, OR) combining MeSH and non-MeSH terms. No filters were applied for date or language.

Two independent reviewers (M.A.L.V and F.S.R) performed the search, study selection, and data extraction. Any disagreements were resolved by a third author. A manual search of references from selected articles was also conducted.

PubMed: (((fatigue[Title/Abstract]) OR (fracture resistance[Title/Abstract])) OR (strength[Title/Abstract])) AND (implantoplasty)[Title/Abstract]

Embase: (‘fatigue’/exp OR fatigue OR (cyclic AND (‘loading’/exp OR loading)) OR ((‘fracture’/exp OR fracture) AND (‘resistance’/exp OR resistance)) OR ‘strength’/exp OR strength) AND (‘implantoplasty’/exp OR implantoplasty)

Scopus: (‘fatigue’/exp OR fatigue OR (cyclic AND (‘loading’/exp OR loading)) OR ((‘fracture’/exp OR fracture) AND (‘resistance’/exp OR resistance)) OR ‘strength’/exp OR strength) AND (‘implantoplasty’/exp OR implantoplasty) (((fatigue) OR (fracture resistance[Title/Abstract])) OR (strength[Title/Abstract])) AND (implantoplasty)[Title/Abstract]

Web of Science: ((((TS = (fatigue)) OR TS = (cyclic loading)) OR TS = (fracture resistance)) OR TS = (strength)) AND TS = (implantoplasty)

### 2.6. Study Selection

After removing duplicates with Rayyan (https://rayyan.ai/cite, accessed on 21 August 2025), two independent reviewers selected the studies based on title and abstract. Full-text articles were then assessed, and studies not meeting the inclusion criteria, such as those not evaluating implant resistance or without implantoplasty, were excluded.

### 2.7. Data Extraction

Extracted variables included author, year, sample size, implant brand and material, implant diameter and length, prosthetic connection type, abutment details, torque, ISO standards, fixation materials, use of protective hemispherical cap, implantoplasty length, technique, burs and equipment used, polishing instruments, magnification, testing machine, chewing simulator, compression test, load cell, cyclic speed, measurement software, test temperature, SEM microscope used, fracture force and fatigue results. Microsoft Excel was used for data management and collection.

### 2.8. Risk of Bias

Risk of bias assessment followed the QUIN tool (risk of bias tool for assessing in vitro studies). Of the 17 in vitro studies evaluated, the result is of medium risk of bias, according to the QUIN evaluation tool, and its rating scale. Risk of Bias is shown in Table 1.

## 3. Results

### 3.1. Development of the Study Selection

**Figure 1:**
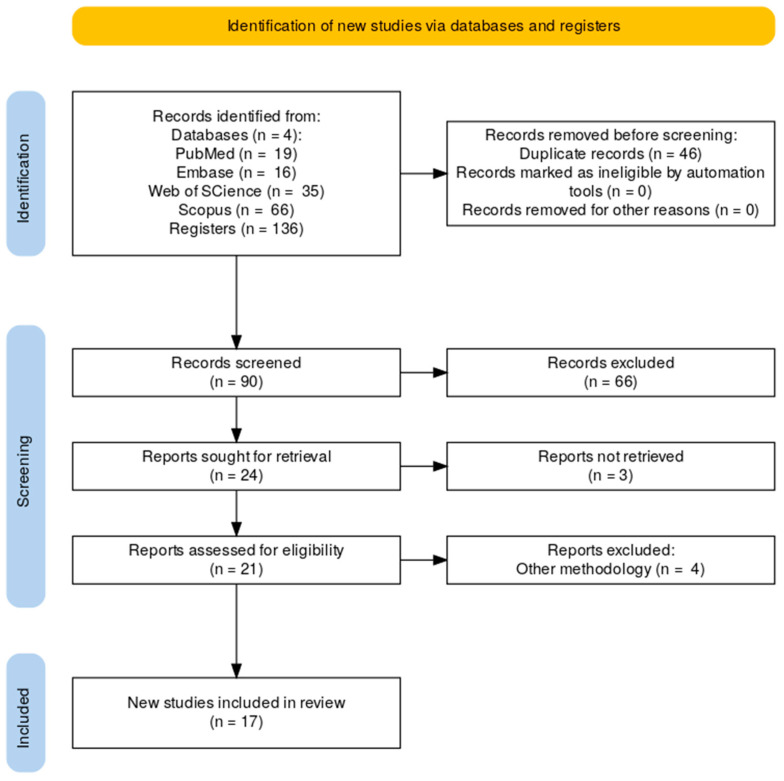
PRISMA Flow Diagram.

The search was conducted in March 2025 and identified 136 articles: 19 in PubMed, 16 in Embase, 35 in Web of Science, and 66 in Scopus. After removing duplicates, 90 articles remained. After screening titles and abstracts, 66 studies were excluded. A total of 21 articles were selected for full-text review, and 4 were excluded for not meeting inclusion criteria, such as not evaluating implant resistance or lacking implantoplasty procedures (Figure 1).

The Meta-analysis was not performed due to the heterogeneity of the studies and the differences in dental implant designs across the studies.

### 3.2. Quality Assesment

Seventeen studies have medium quality based on the QUIN evaluation criterion used for in vitro studies, the scores thus obtained were used to grade the in vitro study as high, medium, or low risk (>70% = low risk of bias, 50% to 70% = medium risk of bias, and <50% = high risk of bias), due to the type of studies there is no randomization of the sample or a clear explanation of the sample calculation, as well as blinding the research advisor (see Table 1).

### 3.3. Study Characteristics

Sample sizes in the selected studies ranged from 18 to 315 implants. Brands included Straumann [18,19], TRI Vent implants [20,25], Biomimetic Ocean, Avinent [21,22,25,26,28], Implacil-Bortoli [23], Neodent [8], Klockner Implant System [27,33], Astra Tech Dentsply Sirona [30], and Conelog [31]. The prosthetic platforms used were external hexagon [8,22,23,26,27], internal hexagon [6,9,19,23,24,25,28,29,31,32], and conical connection [21,23,30]. All studies adhered to ISO 14801 standards [18] (see Table 2).

Implantoplasty lengths ranged from 1.5 mm [30], 3 mm [19,24,26,30,31], 4 mm [9], 4.5 mm [31] to 5 mm [6,20,23,25,26,29,32], 6 mm [8,27], and up to 7 mm [26] (Table 3). Implantoplasty was performed with a lathe in studies [6,9,19,23,25], or manually by a clinical expert in studies [8,9,20,22,24,26,27,28,29,30,32].

### 3.4. Fracture Resistance Results

External hexagon implants (Table 4): 4 mm diameter implants showed control group fracture resistance between 773.1 N and 1660 N, and between 478.1 N and 1650 N after implantoplasty. 3.5 mm implants ranged from 548.82 N to 1276.1 N in control groups and 465.95 N to 1211.7 N after implantoplasty. Some studies compared different implantoplasty techniques or evaluated crown-to-implant ratios [22].

Internal hexagon implants (Table 5): used diameters varied, with lengths of 10–11 mm. Fracture resistance ranged from 812 N [9] to 3325 N [20] in control groups, and from 321.7 N [20] to 739 N [9] post-implantoplasty.

Conical connection implants (Table 6): fracture resistance ranged from 348 N [31] to 2296.68 N [30] in controls, and 315.9 N [31] to 2395.3 N [30] after implantoplasty.

### 3.5. Cyclic Loading Results

Some studies [6,19,25,32] assessed implantoplasty after cyclic chewing simulations with predominant values of 2,000,000 cycles (Table 7).

One study [28] showed results after cyclic loading using 5 × 10^6^ cycles at 15 Hz, with decreasing loads in 5% increments (Table 8).

## 4. Discussion

Implantoplasty, when used in conjunction with surgical procedures, can be considered a viable alternative for the treatment of peri-implantitis [6]. Clinical decision-making should take into account the implant diameter and type of prosthetic connection when selecting implantoplasty as an adjunctive therapy, as it can help maintain the health of peri-implant tissues [1,2]. When properly performed with suitable materials, the procedure reduces bacterial plaque accumulation in the cervical region of the implant. However, it may also compromise the screw and abutment, especially considering the crown-to-implant ratio or lever arm effect.

Various implant designs and prosthetic connections—external hexagon, internal hexagon, and conical—of different diameters and lengths were used in the in vitro studies, allowing a broader understanding of implant behavior after undergoing implantoplasty.

Multiple methodologies and instruments have been employed for performing implantoplasty. The study by Costa-Berenguer et al. [8] is one of the most frequently cited, along with those by Tsampli, De Souza Júnior, Sahrmann, and Ramel [9,10,11,12]. These methods range from conventional rotary instrumentation (using carbide burs and high-speed handpieces) to more recent techniques involving ultrasonic tips with abrasive stones. Each method generates different levels of surface roughness and may induce issues such as thermal damage to peri-implant tissues—especially in structurally weakened implants [10,15] as well as inflammatory responses from titanium particle deposition in soft tissues [33], often linked to implant corrosion [16].

All included studies adhered to ISO 14801:2016 standards [18] for dynamic loading tests in dental implants. However, this standard does not account for peri-implantitis conditions, which introduces variability in measurement distances and load distribution, potentially influencing final results.

Material removal during implantoplasty, while beneficial for decontamination, inevitably weakens the implant structure. Narrow implants are more susceptible to mechanical failure post-procedure [19,20,31], while standard-diameter implants tend to retain greater structural resistance. Longer implantoplasty depths are associated with further decreases in mechanical resistance.

With respect to prosthetic connections, resistance increases as the connection becomes more internal. Thus, implants with external hex connections showed the lowest resistance [8,21,22,23,26,27], followed by internal hex [9,20,21,23], with conical connections being the most resistant [21,23,30,31].

Despite the growing body of evidence, no study has quantitatively assessed the amount of titanium lost during implantoplasty or its correlation with mechanical resistance at various preparation depths. Similarly, few studies have conducted cyclic fatigue tests with clear, standardized reporting.

In this study, we identified several limitations. Since the instruments and techniques used to perform implantoplasty are diverse and the time and calibration of the equipment used are parameters that could influence the outcome of implantoplasty or the degree of wear [9,10,11], it would be beneficial to measure the degree of corrosion [33], the amount of titanium released and the temperature rise [15] caused by the wear that could be released. Furthermore, by using in vitro studies, we inherently assume the limitations of each individual study, highlighting the lack of articles that use implants with a similar length, diameter and prosthetic connection, which makes direct comparisons difficult, and variability in their evaluation method. Therefore, it would be valuable for future research to consider conducting a review of clinical studies with several follow-up periods to obtain more reliable and clinically relevant results.

## 5. Conclusions

Implantoplasty decreases the mechanical resistance of dental implants, particularly in narrow-diameter implants. Increased implantoplasty length correlates with reduced implant strength.

Among prosthetic connections, the external hexagon is the most affected by the procedure, followed by the internal hexagon. The conical connection exhibits the highest mechanical resistance.

## Figures and Tables

**Table 1:** Risk of Bias—QUIN tool (quin assessment tool for in vitro studies), Scores for studies are awarded according to the following. Adequately specified = 2; inadequately specified = 1; not specified = 0; not applicable indicates that this category would not be counted.

		CRITERIA														
		1	2	3	4	5	6	7	8	9	10	11	12			
	STUDY	Clearly stated aims/objectives	Detailed explanation of sample size calculation	Detailed explanation of sampling technique	Details of comparison group	Detailed explanation of methodology	Operator details	Randomization	Method of measurement of outcome	Outcome assessor details	Blinding	Statistical analysis	Presentation of results	SCORE	%	Risk of BIAS
1	Bertl, 2021 [19]	2	0	2	2	2	2	0	2	0	0	2	2	16	66.67	Medium
2	Chan, 2013 [20]	2	0	2	2	2	1	0	2	0	0	2	2	15	62.50	Medium
3	Costa-Berenguer, 2018 [8]	2	0	2	2	2	2	0	2	0	0	2	2	16	66.67	Medium
4	Camps—Font, 2020 [21]	2	0	2	2	2	1	0	2	0	0	0	2	13	54.17	Medium
5	Leitao-Almeida, 2020 [22]	2	0	2	2	2	2	0	2	0	0	2	2	16	66.67	Medium
6	Gehrke, 2016 [23]	2	0	2	2	2	2	0	2	0	0	2	2	16	66.67	Medium
7	Dieguez-Pereira, 2021 [24]	2	0	2	2	2	2	0	2	0	0	2	2	16	66.67	Medium
8	Jorio, 2021 [25]	2	0	2	2	2	2	0	2	0	0	2	2	16	66.67	Medium
9	Leitao-Almeida, 2021 [26]	2	0	2	2	2	2	0	2	0	0	2	2	16	66.67	Medium
10	Sivolella, 2021 [27]	2	0	2	2	2	2	0	2	0	0	2	2	16	66.67	Medium
11	Camps—Font, 2023 [28]	2	0	2	2	2	2	0	2	0	0	2	2	16	66.67	Medium
12	Fonseca, 2024 [29]	2	0	2	2	2	2	0	2	0	0	2	1	15	62.50	Medium
13	Goh, 2024 [30]	2	0	2	2	2	2	0	2	0	0	2	2	16	66.67	Medium
14	Graf, 2023 [31]	2	0	2	2	2	2	0	2	0	0	2	2	16	66.67	Medium
15	Shah, 2024 [32]	2	0	2	2	2	2	0	2	0	0	2	2	16	66.67	Medium
16	Stavropoulos, 2023 [6]	2	0	2	2	2	2	0	2	0	0	2	2	16	66.67	Medium
17	Tsampli, 2024 [9]	2	0	2	2	2	2	0	2	0	0	2	2	16	66.67	Medium

**Table 2:** Implants Used in the Studies: Manufacturer, Dimensions, Connection Type, and Prosthetic Platform.

Author/Year	Sample Size (n)	Implant Brand	Titanium Grade	Implant Dimensions	Connection Type	Prosthetic Hex Diameter
Chan, 2013 [20]	32	TRI-Vent implants (TRI Dental Implants)	Not specified	3.75 × 10 mm	Internal hexagon	1.5 mm depth;
				(Narrow); 4.7 × 10 mm (Wide)		2.5 mm hexagon
Gehrke, 2016 [23]	60	Implacil Bortoli	Not specified	4 × 11 mm	External hex;	Not specified
					Internal hex;	
					Morse taper	
Costa-Berenguer, 2018 [8]	20	Titamax Smart Cortical, Neodent, Curitiba, Brazil	Grade 4	4 × 13 mm	External hex	4.1 mm platform, 2 mm screw
Camps-Font, 2020 [21]	48	Biomimetic Ocean^®^, Avinent^®^, Spain	Grade 5	3.5 × 10 mm	External hex;	3.5 mm
					Internal hex;	
					Conical connection	
Shah, 2024 [32]	28	Roxolid Bone Level Implant (Straumann)	TiZr (85% Ti, 15% Zr)	4.1 × 10 mm	Not specified	4.1 mm
Camps-Font, 2023 [28]	20	Biomimetic Ocean^®^, Avinent^®^, Spain	Grade 5	3.5 × 10 mm	Internal hex	Not specified
Fonseca, 2024 [29]	120	Klockner Implant System, Andorra	Grade 3	N/A	Not specified	Not specified
Dieguez-Pereira, 2021 [24]	315	Klockner Essential Cone, Andorra	—	3.5 × 10 mm;	Internal hex	2.7–3.4 mm
				4 × 10 mm		
Goh, 2024 [30]	80	AstraTech EV, Dentsply Sirona, USA	Grade 4	4.2 × 13 mm	Conical connection	Not specified
Stavropoulos, 2023 [6]	N/A	Straumann AG, Basel, CH	Grade 4 Ti	3.3 × 10 mm	Internal hex	Not specified
			Ti and Zr			
Jorio, 2021 [25]	30	TRI-Vent Bone-Level Implant, TRI Dental Implants, Switzerland	Grade 5	4.1 × 11 mm	Not specified	Not specified
Leitao-Almeida, 2020 [22]	48	Ocean E.C., Avinent, Spain	Grade 5	3.5 × 15 mm	External hex	Not specified
Leitao-Almeida, 2021 [26]	32	Ocean E.C., Avinent Implants System S.L., Santpedor, Spain	Grade 5	3.5 × 15 mm	External Hex	Not specified
Bertl, 2021 [19]	112	Straumann AG, Switzerland	Ti	3.3 × 10 mm	Internal hex	Not specified
			TiZr	4.1 × 10 mm		
Graf, 2023 [31]	90	Conelog SCREW-LINE, Camlog, Switzerland	N/A	3.3 × 13 mm	Internal hex	Not specified
				3.8 × 13 mm		
				4.3 × 13 mm		
Sivolella, 2021 [27]	18	Osseotite^®^ Hybrid, Zimmer Biomet, USA	—	4 × 13 mm	External hex	Not specified
Tsampli, 2024 [9]	30	Premium, Medentis Medical, Germany	Grade 4	4.1 × 10 mm	Internal hex	Not specified

**Table 3:** Implantoplasty: Length, Technique, Operator, and Instruments Used.

Author/Year	Implantoplasty Height/Length	Operator	Instruments Used	Handpieces Used	Polishing Instruments	Magnification Used
Chan, 2013 [20]	5 mm	Periodontist	Diamond bur 30–15 µm oval (Henry Schein)	15,000 rpm	Arkansas stones	×2.5 (Design for Vision)
					Fine silicone polishers (Henry Schein)	
Gehrke, 2016 [23]	5 mm	Machined	Tungsten carbide conical burs on machine (Model BV-20 Ferrari), wear rate 0.050 µm/min without irrigation	20,000 rpm	Not specified	Not specified
Costa-Berenguer, 2018 [8]	6 mm	Expert clinician	Tungsten carbide oval burs (H379 314 023; Komet Dental, Germany)	High-speed handpiece (SUPER torque 660, KaVo, Germany) with copious irrigation	Two-step silicone polishers (9618 314 030 and 9608 314 030, Komet)—new set per implant	×2.5 (Heine dental loupes)
Camps-Font, 2020 [21]	5 mm	Experienced clinician	Followed Costa-Berenguer protocol	High-speed handpiece (GENTLE silence LUX 8000B, KaVo)	Followed Costa-Berenguer protocol	×2.8 (Galilean HD, ExamVision)
Shah, 2024 [32]	5 mm	Expert clinician	Ball-shaped diamond burs: coarse (107 µm), medium (46 µm), fine (25 µm)—Komet	Electric high-speed handpiece (Bien-Air) at 200,000 rpm	Silicone polishers at 20,000 rpm (brown, green, and supergreen; Shofu Corp.)	Not specified
Camps-Font, 2023 [28]	5 mm	Expert clinician	Costa-Berenguer protocol	High-speed handpiece: Gentle Silence 8000B (KaVo)	Costa-Berenguer protocol	×2.8 (Galilean HD, ExamVision)
Fonseca, 2024 [29]	Not specified	Not specified	Fine-grain tungsten carbide bur (H379.314, KOMET)	High-speed turbine (GENTLE silence LUX 8000B, KaVo)	Coarse to fine polishing burs; Carbon polishers (9608.314.030 and 9618.314.030, KOMET)	Not specified
Dieguez-Pereira, 2021 [24]	3 mm (bone level), 1.5 mm (tissue level)	Expert clinician	Costa-Berenguer protocol; 3 mm of exposed threads removed with oval tungsten carbide bur (H379 314 023; Komet Dental, Lemgo, Germany)	High-speed turbine (Panamax 2, NSK)	Costa-Berenguer; the surface was polished according to that methodology using two silicone polishers (9618 314 030 and 9608 314 030; Komet Dental). Additionally, controlled reduction was performed using an industrial machine (Deco 2000, Tornos Technologies Iberica, Granollers, Spain).	×2.5 (Zeiss)
	3 mmm tissue level and bone level					
Goh, 2024 [30]	3 mm	Single operator	Tungsten carbide burs (Meisinger, Germany)	High-speed handpiece (Dentsply Sirona) at 40,000 rpm with illumination and irrigation	Tungsten carbide burs (Meisinger)	×2.5 (ZEISS EyeMag Smart)
	5 mm					
Stavropoulos, 2023 [6]	5 mm apically from (a) the implant neck in bone-level implants and (b) from the machined roughness in tissue-level (TL) implants. The implant diameter was reduced by 0.13 mm in bone-level implants and 0.15 mm in tissue-level implants.	High-precision Tornos (Schaublin, 180-CCN—BL 3267, SCHAUBLIN MACHINES SA, Bévilard, CH, USA)	Not specified	Not specified	Not specified	Not specified
Jorio, 2021 [25]	5 mm	A single right-handed operator, trained and calibrated according to the protocols of references.	Based on prior studies (Ramel, Sahrmann, Chan)	According to cited studies	According to cited studies	×2.7 (Galilean HD, ExamVision)
Leitao-Almeida, 2020 [22]	7.5 mm	Experienced surgeon	Oval tungsten carbide bur (H379 314 023; Komet Dental, Lemgo, Germany)	High-speed handpiece (Bora blackline LED, Bien-Air)	Two-step silicone polishers (9618 314 030 and 9608 314 030; Komet)	×2.8 (Galilean HD, ExamVision)
Leitão-Almeida, 2021 [26]	3 mm and 7.5 mm	Experienced surgeon	Costa-Berenguer; Oval carbide bur ((H379 314 023; Komet Dental, Lemgo, Germany)	Not specified	Two-step silicone polishers (9618 314 030 and 9608 314 030; Komet Dental, Lemgo, Germany)	×2.8 (Galilean HD, ExamVision)
Bertl, 2021 [19]	Extended implantoplasty 3 mm apically from the implant neck in bone-level implants and from the machined surface in tissue-level implants. The diameter was reduced by 0.13 to 0.16 mm (i.e., narrow BL: 0.13 mm; narrow TL: 0.15 mm; regular BL: 0.14 mm; regular TL: 0.16 mm).	Computer-controlled lathe (Tornos-Schaublin, 180-CCN—BL 3267, SCHAUBLIN MACHINES SA, Bévilard, Switzerland)	Not specified	Not specified	Not specified	Not specified
Graf, 2023 [31]	1.5 mm	Not specified	Not specified	Not specified	Not specified	Not specified
	3.0 mm					
	4.5 mm					
Sivolella, 2021 [27]	6 mm	Experienced clinician	Two oval tungsten carbide burs (H379.310.023 and H379UF.310.023, Komet Dental, Lemgo, Germany)		Both groups were treated with Arkansas stones (Dura-White Stones FL2 FG 0.244, Shofu, Kyoto, Japan) (BUR + A and SONIC + A, respectively).	Not specified
			A sequence of two torpedo-shaped diamond burs (SF878K.000.018 and SF8878K.000.018, Komet Dental)	Air scaler (SF1LM, Komet Dental) (SONIC)		
Tsampli, 2024 [9]	4 mm	First phase: Universal testing machine (Z005, Zwick/Roell, Ulm, Germany) with 3D-printed elements holding either the air scaler or the NSK surgical handpiece. A sequence of two torpedo-shaped diamond burs (SF878K.000.018 and SF8878K.000.018, Komet Dental) was used.	A sequence of two torpedo-shaped diamond burs (SF878K.000.018 and SF8878K.000.018, Komet Dental). Two oval tungsten carbide burs (H379.310.023 and H379UF.310.023, Komet Dental, Lemgo, Germany), used with progressively finer polishers and abrasives mounted on the NSK surgical handpiece (X-SG 93, 1:3 ratio, NSK, Funck, dental-medizin, Heidelberg, Germany). Only one bur was used per implant (no reuse), operating at 60,000 rpm.	Not specified	Not specified	Not specified
		Second hase: dentist	AIRSCALER group: The implants were treated using precision tungsten carbide tips (diameter: 2.5 mm, grade: G10, material: TC2, and hardness: HV 1400–1500) soldered to a stainless steel shaft. These tips were custom-made on a precision machine at the University of Heidelberg as part of a self-funded research initiative. The air scaler (SONICflex 2003L, KAVO Dental, Biberach/Riß, Germany) operated at 4.2 bar pressure. The active part of the tips had a diameter of 2.5 mm. Five passes were performed, removing 0.1 mm of material.			

**Table 4:** Fracture Test Results of External Hexagon Implants.

Author/Year	Sample Size	Implant	Dimensions	Prosthetic Connection	Testing Machine	Chewing Simulator	Compression Test Speed	Cyclic Speed	Measurement Software	Control Group (SD) [N]	Fracture Resistance (SD) [N]
Gehrke, 2016 [23]	60	Implacil De Bortoli	4 × 11 mm	External hex	Universal testing machine (AME-5 kN)	N/A	1 mm/min	N/A	N/A	HE = 773.1 (13.16)	487.1 (93.72)
Costa-Berenguer, 2018 [8]	20	Titamax Smart Cortical, Neodent	4 × 13 mm	External hex	Universal servo-hydraulic mechanical testing machine (BIONIX 370, MTS)	N/A	1 mm/min	N/A	N/A	880 (193.7)	896 (121.1)
Camps-Font, 2020 [21]	48	Biomimetic Ocean^®^, Avinent^®^	3.5 × 10 mm	External hex	MTS Bionix 370 Load Frame	N/A	1 mm/min	N/A	TestStar II^®^	1211.90 (89.85)	HE = 873.11 (92.37)
Leitao-Almeida, 2020 [22]	48	Ocean E.C., Avinent^®^	3.5 × 15 mm	External hex	Universal servo-hydraulic mechanical testing machine (MTS Bionix 370)	N/A	1 mm/min	N/A	MTS Flextest 40	2:1 = 1276.16 (169.75)	1211.70 (281.64)
										2.5:1 = 815.22 (185.58)	621.68 (186.28)
										3:1 = 606.55 (111.48)	465.95 (68.57)
Leitão-Almeida, 2021 [26]	32	Ocean E.C., Avinent^®^	3.5 × 15 mm	External hex	Universal mechanical testing machine (MTS Bionix 370)	N/A	N/A	N/A	MTS Flextest 40	C = 854.37 (195.08)	752.12 (186.13)
										C = 548.82 (80.02)	593.69 (111.07)
Sivolella, 2021 [27]	18	Osseotite^®^ Hybrid, Zimmer Biomet	4 × 13 mm	External hex	MTS Acumen 3 Electrodynamic Test System	N/A	1 mm/min	N/A	MTS Testsuite	166,000 (38,000)	Bur = 151,000 (17,000);
											Sonic = 165,000 (24,000)

**Table 5:** Fracture Test Results of Internal Hexagon Implants.

Author/Year	Sample Size	Implant	Dimensions	Prosthetic Connection	Testing Machine	Chewing Simulator	Compression Test Speed	Cyclic Speed	Measurement Software	Control Group (SD) [N]	Fracture Resistance (SD) [N]	Fracture Resistance in Machined Implants
Chan, 2013 [20]	32	TRI-Vent implants (TRI Dental Implants)	3.75 × 10 mm (N)	Internal hex	Universal testing machine (Instron 5565)	N/A	0.5 mm/min	N/A	Merlin Software (Instron Corp.)	C = 3325 (20.7)	W = 430, 4 (26.8)	N/A
			4.7 × 10 mm (W)								N = 321, 7(214)	
Gehrke, 2016 [23]	60	Implacil De Bortoli	4 × 11 mm	Internal hex	Universal testing machine (model AME-5 kN, Tecnica Industrial Oswaldo Filizola)	N/A	1 mm/min	N/A	N/A	HI = 829.4	495.7 (85.24)	N/A
Camps-Font, 2020 [21]	48	Biomimetic Ocean^®^, Avi- nent^®^ Implant System, Santpedor, Spain	3.5 × 10 mm	Internal hex	MTS Bionix 370 Load Frame universal servo-hydraulic mechanical testing machine (MTS^®^, Eden Prairie, USA)	N/A	1 mm/min	N/A	TestStar II^®^ software (MTS^®^, Eden Prairie, USA)	918.41 (97.19)	HI = 661.29 (58.03)	N/A
Tsampli, 2024 [9]	30	Premium, Medentis Medical, Bad Neuenahr- Ahrweiler, Germany	4.1 × 10 mm	Internal hex	universal testing device (Z005, Zwick/Roell, Ulm, Germany).	N/A	1 mm/min	N/A	N/A	812 (30)	N/A	Bur = 665 (26)
												Airscaler = 739 (34)

**Table 6:** Fracture Test Results of Conical Connection Implants.

Author/Year	Sample Size	Implant	Dimensions	Prosthetic Connection	Testing Machine	Chewing Simulator	Compression Test Speed	Cyclic Speed	Measurement Software	Control Group (SD) [N]	Fracture Resistance (SD) [N]
Gehrke, 2016 [23]	60	Implacil De Bortoli	4 × 11 mm	Morse taper	Universal testing machine (model AME-5 kN, Tecnica Industrial Oswaldo Filizola)	N/A	1 mm/min	N/A	N/A	CM = 898.1 (19.25)	717.6 (77.25)
Camps-Font, 2020 [21]	48	Biomimetic Ocean^®^, Avi- nent^®^ Implant System, Santpedor, Spain	3.5 × 10 mm	Conical connection	MTS Bionix 370 Load Frame universal servo-hydraulic mechanical testing machine (MTS^®^, Eden Prairie, USA)	N/A	1 mm/min	N/A	TestStar II^®^ software (MTS^®^, Eden Prairie, USA)	1058.67 (114.05)	CC = 747.32 (90.05)
Goh, 2024 [30]	80	AstraTech Implant System EV, Dentsply Sirona	4.2 × 13 mm	Conical connection	Universal mechanical testing machine (Instron 3369, Instron Ltd., High Wycombe, UK)	N/A	1 mm/min	It is calculated by subtracting 0.5 from the fracture load, and the elastic limit is then determined	N/A	3 mm = 2466.64 (173.36)	3 mm = 2349.18 (142.51);
										5 mm = 1797.76 (119.86)	5 mm = 1431.84 (1887.78);
										D.V. 3 mm = 2659.06 (123.19);	D.V. 3 mm = 2395.32 (144.99);
										D.V. 5 mm = 2296.78 (147.46)	D.V. 5 mm = 1866.29 (164.26)
Graf, 2023 [31]	90	Conelog implants (CONELOG SCREW-LINE implant, Promote plus, Camlog Biotechnologies AG, Basel, Switzerland)	3.3 × 13 mm	Conical connection	Zwick UPM 1445; Zwick GmbH & Co. KG, Ulm, Germany)	Chewing simulator (CS-4 chewing simulator; SD Mechatronik, Feldkirchen-Westerham, Germany)	0.5 mm/min	1,200,000 cycles at 50 N	N/A	D3.3 = 348.3 (50.3)	D3.3–15 = 382.1 (59.2)
			3.8 × 13 mm							D3.8 = 507.9 (40.7)	D3.3–30 = 347.0 (35.7);
			4.3 × 13 mm							D4.3 = 690.1 (53.4)	D3.3–45 = 315.9 (30.9)
											D3.8–15 = 531.4 (36.2)
											D3.8–30 = 514.5 (40.8)
											D3.8–45 = 477.9 (26.3)
											D4.3–15 = 710.1 (38.2)
											D4.3–30 = 697.9 (65.2)
											D4.3–45 = 662.2 (45.9)

**Table 7:** Fracture Results of Implants after Cyclic Loading.

Author/Year	Sample Size	Implant	Dimensions	Prosthetic Connection	Testing Machine	Chewing Simulator	Compression Test Speed	Cyclic Speed	Measurement Software	Control Group (SD) [N]	Fracture Resistance (SD) [N]	Fracture Resistance in Maquinated Implants	Results After Cycles
Shah, 2024 [32]	28	Roxolid Bone Level Implant Regular CrossFit SLA; Institut Straumann AG)	4.1 × 10 mm	Internal hex	4204 tensile tester (Instron)	N/A	1 mm/min	2,000,000 cycles at 2 Hz frequency with a compressive load of 250 N by a plastic ball. The 2,000,000 cycles used in this study were approximately equivalent to 2 years of function	N/A	No cycles = 1465.2 (86.4)	No cycles = 1299.3 (123.8);	N/A	N/A
										Cycles = 1480.7 (64.1)	Cycles = 1252.1 (85.7)	N/A	
Stavropoulos, 2023 [6]	N/A	Straumann AG	3.3 × 10 mm	Internal hex	Universal testing machine (Instron 4465; Instron Co., Ltd.	Pre- load device to sim- ulate mastication.	1 mm/min	All implants were subjected to 2,000,000 cycles of loading with 23–226 N at 2 Hz, at room temperature and in a moist environment	N/A	BL = 435.2–550.6 N	BL = 400.9–495.3 N		N/A
										TL = 389.5–495.8 N	TL = 353.0 N		
Jorio, 2021 [25]	30	TRI- Vent BoneLevel Dental Implants Int. AG, Switzerland	4.1 × 11 mm	Internal hex	Zwick. 1445 RetroLine, Zwick,	Computer- controlled masticator. During this experiment, suffered thermocycling.	N/A	1.2 million cycles, 49 N, thermal cycling	N/A	C(cycles) = 2299 (127)	IP1 = 1642 (51)		After 1.2 million cycles and 10,000 temperature changes, no fractures observed
											IP2 = 1792 (47)		
											IP3 = 1777 (49)		
										C(w/cycles) = 2724 (70)			
Bertl, 2021 [19]	112	(Institut Straumann AG, Basel, CH)	3.3 × 10 mm (Narrow: bone level and tissue level)	Internal hex	Universal testing machine (Instron 4465, Instron Co., Ltd., Norwood, MA, USA)	MTI Engineering. To simulate mastication. Implants were loaded for 2,000,000 cycles at 2 Hz. The mean max- imum failure strength of 3 narrow diameter Ti TL implants not subjected to IP.	1 mm/min	2,000,000 cycles at 2 Hz with 23 to 226 N ambient temperature, corresponding to 10 and 50% of the failure of 3 narrow implants without IP	N/A	Narrow; B.L; Ti = 564.03 (529.13–621.22		Narrow; B.L; Ti = 540.41 (518.13–541.75)	N/A
										Narrow; B.L; TiZr = 569.91 (530.33–577.32)		Narrow; B.L; TiZr = 477.32 (459.34–506.72)	
										Narrow; T.L; Ti = 472.76 (462.02–481.08)		Narrow; T.L; Ti = 363.90 (362.56–366.85)	
										Narrow; T.L; TiZr = 476.78 (473.90–481.08)		Narrow; T.L; TiZr = 398.26 (397.45–408.86)	
			4.1 × 10 mm (Regular: Bone level and tissue level)							Regular, B.L; Ti = 938.53 (921.07–982.90)		Regular, B.L; Ti = 870.34 (857.72–876.38)	
										Regular, B.L; TiZr = 986.58 (967.45–1000.34)		Regular, B.L; TiZr = 863.90 (848.06–882.43)	
										Regular, T.L; Ti = 785.24 (771.82–786.18)		Regular, T.L; Ti = 695.04 (689.27–705.64)	
										Regular, T.L; TiZr = 794.10 (779.74–795.04)		Regular, T.L; TiZr = 713.56 (693.69–726.98)	

**Table 8:** Implantoplasty Results under Cyclic Loading.

Author/Year	Sample Size	Implant	Dimensions	Prosthetic Connection	Testing Machine	Chewing Simulator	Compression Test Speed	Cyclic Speed	Measurement Software	Results After Cycles	Control After Cycles
Camps-Font, 2023 [28]	20	Biomimetic Ocean^®^, Avinent^®^ Implant System, Santpedor, Spain	3.5 × 10 mm	Internal hex	MTS Bionix 370, MTS^®^, Eden Prairie, MN, USA	N/A	N/A	5 × 10^6^ cycles, 15 Hz	TestStar II^®^ software (MTS^®^, Eden Prairie, MN, USA)	95%: 628 N, 5 × 10^6^–102,360	80% = 735 N; 36,364–66,690
										90% = 628 N, 279,251–5 × 10^6^	70% = 643 N; 38,830–68,519
										85% = 562 N, 318,799–5 × 10^6^	65% = 597 N; 112,481–85,644
										80% = 529 N, 5 × 10^6^	60% = 551 N; 5 × 10^6^
